# A Single Nucleotide Polymorphism in Human APOBEC3C Enhances Restriction of Lentiviruses

**DOI:** 10.1371/journal.ppat.1005865

**Published:** 2016-10-12

**Authors:** Cristina J. Wittkopp, Madison B. Adolph, Lily I. Wu, Linda Chelico, Michael Emerman

**Affiliations:** 1 Department of Microbiology, University of Washington, Seattle, Washington, United States of America; 2 Division of Human Biology, Fred Hutchinson Cancer Research Center, Seattle, Washington, United States of America; 3 Division of Basic Sciences, Fred Hutchinson Cancer Research Center, Seattle, Washington, United States of America; 4 Department of Microbiology and Immunology, College of Medicine, University of Saskatchewan, Saskatoon, Canada; University of Illinois at Chicago College of Medicine, UNITED STATES

## Abstract

Humans express seven human APOBEC3 proteins, which can inhibit viruses and endogenous retroelements through cytidine deaminase activity. The seven paralogs differ in the potency of their antiviral effects, as well as in their antiviral targets. One APOBEC3, APOBEC3C, is exceptional as it has been found to only weakly block viruses and endogenous retroelements compared to other APOBEC3s. However, our positive selection analyses suggest that APOBEC3C has played a role in pathogen defense during primate evolution. Here, we describe a single nucleotide polymorphism in human APOBEC3C, a change from serine to isoleucine at position 188 (I188) that confers potent antiviral activity against HIV-1. The gain-of-function APOBEC3C SNP results in increased enzymatic activity and hypermutation of target sequences when tested *in vitro*, and correlates with increased dimerization of the protein. The I188 is widely distributed in human African populations, and is the ancestral primate allele, but is not found in chimpanzees or gorillas. Thus, while other hominids have lost activity of this antiviral gene, it has been maintained, or re-acquired, as a more active antiviral gene in a subset of humans. Taken together, our results suggest that APOBEC3C is in fact involved in protecting hosts from lentiviruses.

## Author Summary

The human *APOBEC3* gene family consists of seven cytidine deaminases that mutate viral genomes. Compared to the other six human APOBEC3s, APOBEC3C has poor activity against viruses as well as endogenous retroelements, and its function remains poorly understood. Here, we report that although most humans express a version of APOBEC3C that only weakly blocks HIV, there is a polymorphism found in African populations that drastically enhances its anti-HIV activity. Furthermore, we demonstrate that the more active variant more efficiently deaminates cytidines *in vitro* and, in contrast to the common variant, forms dimers in solution. This polymorphism is absent in other hominids (chimpanzees and gorillas) but reverted or was maintained in some humans. Thus, while many humans have a “hole” in their innate defense against retroviruses, an ancient human polymorphism has restored this antiviral gene in some populations.

## Introduction

The *APOBEC3* locus encodes seven cytidine deaminase proteins that inhibit endogenous retroelements, lentiviruses such as HIV-1, and other viruses [1]. The *APOBEC3* locus arose through duplication events on chromosome 22[2] of cytidine deaminase domains, resulting in single domain *APOBEC3*s (*APOBEC3A*, *APOBEC3C*, and *APOBEC3H*) and double-domain *APOBEC3* genes (*APOBEC3B*, *APOBEC3D*, *APOBEC3F*, and *APOBEC3G*). In order for APOBEC3 proteins to restrict lentiviruses such as HIV-1, they are packaged into virions, brought to a target cell, and deaminate cytidines on ssDNA during reverse transcription, resulting in cytidine to uracil mutations in the viral genome. APOBEC3 proteins exert selective pressure on primate lentiviruses, which have evolved to encode a protein, Vif, which targets APOBEC3 proteins for proteasomal degradation.

Over long evolutionary time-periods, Vif-mediated antagonism of APOBEC3 proteins in populations infected with a lentivirus selects for polymorphisms in the population that have acquired mutations in *APOBEC3* that allow for escape from Vif but maintenance of antiviral activity [3]. Lentiviruses, in turn, select for Vif alleles that target these APOBEC3 variants, leading to further adaptive evolution of *APOBEC3* genes through selection for mutations that allow that host to evade viral infections. As such, enrichment of the rate of nonsynonymous mutations (dN) compared to the rate of synonymous mutations (dS), called positive selection (defined as dN/dS>1), is a common signature of antiviral genes [3]. *APOBEC3* genes involved in blocking viral replication are expected to exhibit signatures of positive selection. Specifically, APOBEC3s involved in lentiviral restriction should have signatures of positive selection at the Vif:APOBEC3 interface [4].

There is considerable variation in the antiviral activity of each of the seven human APOBEC3 paralogs. APOBEC3G potently inhibits *vif*-deleted-HIV-1 (Δ*vif*) [5]. Human APOBEC3D, APOBEC3F, and APOBEC3H also inhibit HIV-1 (Δ*vif*), but to a lesser extent than APOBEC3G [5–8]. In contrast, APOBEC3A and APOBEC3B do not potently block HIV infection of T cells [5–7, 9], which are the primary target of HIV (although a target-cell effect has been reported in monocytes for APOBEC3A) [10]. Instead, APOBEC3A and APOBEC3B drastically inhibit replication of endogenous retroelements such as LINE-1 elements, as well as some DNA viruses [11–16]. In studies that compare the ability of the seven human APOBEC3s to restrict lentiviruses and endogenous retroelements, the only APOBEC3 that has weak activity against both lentiviruses and endogenous retroelements is APOBEC3C [5, 6, 12, 16–22].

For another *APOBEC3* gene, *APOBEC3H*, the most common human variant does not block HIV infection although other human haplotypes exist that potently restrict lentivirus replication [23]. In fact, one haplotype of APOBEC3H restricts HIV-1(Δ*vif*) nearly as potently as APOBEC3G [23] and has been shown to impact clinical outcomes in HIV-1+ patients [24–26]. Thus, we considered the possibility that while the common human haplotype of *APOBEC3C* encodes a protein with little antiviral activity, other variants of *APOBEC3C* may, in fact, encode more potent anti-lentiviral proteins. Moreover, the Vif protein of HIV-1 targets human APOBEC3C for proteosomal degradation [27]. In addition, APOBEC3C mRNA is highly expressed in the major HIV-1 target cells, activated T cells[28]. Thus, the high expression of APOBEC3C in HIV target cells and the antagonism of APOBEC3C by HIV-1 Vif are consistent with the hypothesis that APOBEC3C may have an overlooked role in combating lentivirus infection.

In this study, we found that *APOBEC3C* has evolved under positive selection in primates, in a manner that suggests that APOBEC3C has played a role in blocking primate lentiviruses. This provided motivation to determine if there are naturally occurring variants of APOBEC3C that potently block lentivirus replication. In humans, only one APOBEC3C coding variant is present at a frequency above 1% and this is a serine to isoleucine change at position 188, here called APOBEC3C I188 [29]. We show that the polymorphism APOBEC3C I188 is present at about 10% frequency in diverse populations throughout Africa, and thus did not recently arise in a particular subpopulation of humans, but is an ancient allele that has likely been circulating in humans for much of human history. Moreover, we show that the APOBEC3C I188 single nucleotide polymorphism (SNP) has about 10-fold more potent anti-lentiviral activity than the common human APOBEC3C variant and has greater *in vitro* cytidine deaminase specific activity. The greater activity of APOBEC3C I188 in turn correlates with its ability to dimerize. Moreover, construction of a forced dimer of APOBEC3C S188 also gains enhanced antiviral activity to a level comparable to APOBEC3G. We also show that the APOBEC3C I188 allele is likely the ancestral state since all sequenced Old World monkeys and some great apes carry isoleucine at position 188. However, gorillas, chimpanzees and most humans carry the S188, the apparent loss of function allele. Taken together, our results suggest that APOBEC3C is involved in protecting hosts from lentiviruses, and we speculate that some humans may be afforded some level of additional protection from lentiviruses by a more active antiviral version of this protein.

## Results

### *APOBEC3C* has evolved under positive selection in primates, suggesting an ancient role in protection from pathogens

**Fig 1:**
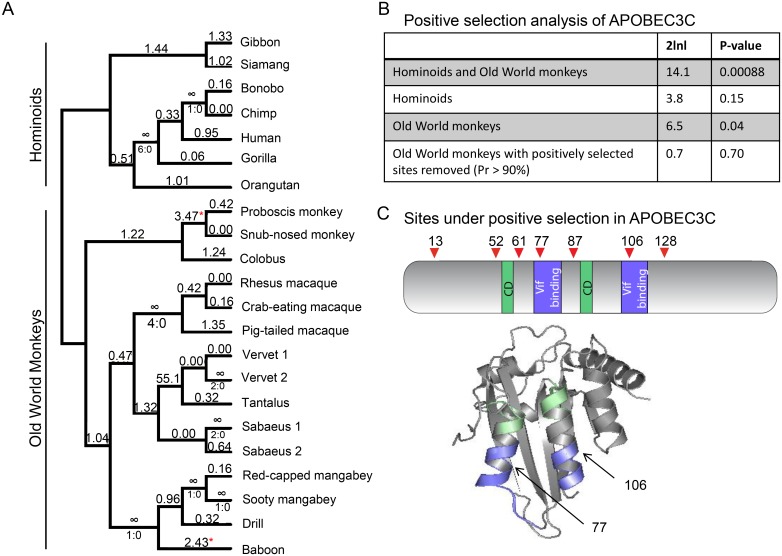
APOBEC3C is rapidly evolving in primates. (A) Twenty-two primate APOBEC3C coding sequences were obtained by PCR or from the NCBI sequence database. A phylogeny of APOBEC3C, indicating the branch analysis results of the positive selection tests. The ratio of rate of nonsynonymous changes (dN) and the rate of synonymous changes (dS) that occurred along each branch are shown above each branch. For dN/dS values = ∞, the total number of non-synonymous changes (N) and synonymous changes (S) are shown (N:S) below the branch. The red asterisks mark the branches where the dN/dS is significantly greater than 1 across the entire gene. (B) Maximum likelihood tests for positive selection, with 2lnl values indicating twice the log difference between the model that allows for positive selection (M8) and the model that does not allow for positive selection (M8a), as well as a P-value to indicate whether the M8 model better fits the data than the M8a model. (C) Sites under positive selection in APOBEC3C are shown in a cartoon diagram, comparing these sites to the Vif binding domain and the cytidine deaminase enzymatic domain (CD). Red triangles depict sites with a posterior probability >0.99 (red triangle). The structure of APOBEC3C[31] is represented, with the Vif binding domain[31] shown in blue. Two of the seven positively selected sites (PP > 0.99) overlap with this domain, are shown with arrows. The cytidine deaminase domain is shown in green.

In studies that compare the antiviral activity of the seven APOBEC3 paralogs, APOBEC3C consistently has poorer restriction activity than the other paralogs [5, 6, 18, 20, 21]. However, we reasoned that if APOBEC3C is in fact a *bona-fide* restriction factor then we would expect that the gene has an evolutionary signature of positive selection [3]. We performed positive selection analyses of twenty-two *APOBEC3C* sequences derived from eighteen primate species with sequences representing diverse clades of catarrhines, a subdivision of primates including old world monkeys and apes (Fig 1A). Among these, multiple sequences were obtained from African green monkeys, because we chose to include three subspecies (vervet, tantalus, and sabeus). The sequences were aligned and tests for positive selection were conducted using maximum likelihood ratio tests comparing M8 (a model that allows positive selection across the gene) to M8a (a model that disallows positive selection). Our results indicate *APOBEC3C* shows a gene-wide signature of positive selection (p<0.0008) (Fig 1B).

We next analyzed individual lineages to determine which branches of the *APOBEC3C* tree have signatures of positive selection. Branch analysis identified two branches with statistically significant signatures of positive selection, both in Old World monkeys (Fig 1A), and while most were not statistically significant, many branches had a dN/dS >1 (Fig 1A). Furthermore, we performed M8 vs M8a analysis of the hominoid and Old World monkey clades of the tree separately, and found that the Old World monkey clade has a statistically significant signature of positive selection (p<0.05) (Fig 1B). We did not see a statistically significant signature of positive selection in the hominoid-only branch (p = 0.15), although this could be due to a smaller sample size (n = 7).

For antiviral genes, sites under positive selection often correlate with sites of interaction with a viral antagonist [30]. APOBEC3C is antagonized by the lentiviral protein Vif and the interface of Vif binding has been extensively mapped [31]. If APOBEC3C is in fact an anti-lentiviral gene, the Vif binding interface may be evolving under positive selection. Therefore, we performed a site-analysis to determine which amino acids are under positive selection across the tree. Our analysis indicated seven sites under positive selection (posterior probability > 99%) (Fig 1C). Next, we mapped the positively selected sites onto the structure of human APOBEC3C and compared these to the Vif interface of APOBEC3C. Of the seven positively selected sites, two of these, residues 106 and 77, are located within the two helices that are targeted by Vif (Fig 1C). Strikingly, residue 106 has been identified as being a critical amino acid for the interaction of APOBEC3C with Vif [27, 31]. Thus, *APOBEC3C* has evolved under selection, gene-wide, as well as at the Vif-binding interface. These results suggest that although the common human APOBEC3C variant does not potently block lentivirus replication, primate *APOBEC3C* may have evolved as an anti-lentiviral protein.

### Human APOBEC3C SNP I188 increases antiviral activity

**Fig 2:**
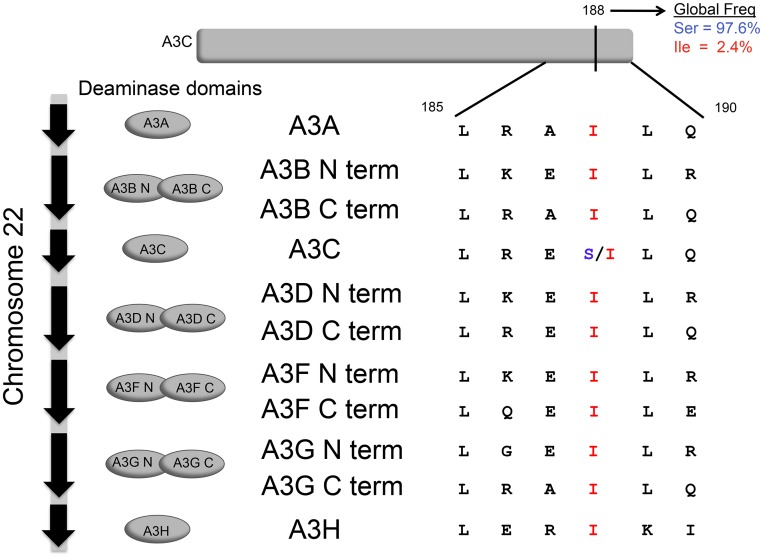
I188 is a SNP in APOBEC3C and is the conserved residue in the other six human APOBEC3 paralogs. Alignment of the seven APOBEC3 proteins, using both deaminase domains (N and C terminal) of the double-domain APOBEC3 proteins (11 domains total). The residue homologous to APOBEC3C in the other ten deaminase domains is conserved as an isoleucine, whereas APOBEC3C is the only domain with a serine at that position. However, human APOBEC3C is polymorphic at that position, with an isoleucine at an allele frequency of 2.4% globally[29].

Because the positive selection analyses suggested an ancient or ongoing role of APOBEC3C in lentiviral restriction (Fig 1), we re-evaluated human polymorphisms in *APOBEC3C* for potential variants with increased activity. There is only one SNP in *APOBEC3C* above 1% frequency globally, and this is a serine to isoleucine change at position 188 [29]. To evaluate the potential significance of this SNP, we aligned this region of *APOBEC3C* to other human *APOBEC3* genes. Strikingly, we found that in contrast to *APOBEC3C*, the other ten *APOBEC3* deaminase domains all encode a conserved isoleucine at the position homologous to APOBEC3C 188 (Fig 2). Thus, the human I188 polymorphism in APOBEC3C actually encodes an amino acid that is highly conserved at this position across human APOBEC3s, while the more common APOBEC3C in the human population has a different amino acid at position 188.

**Fig 3:**
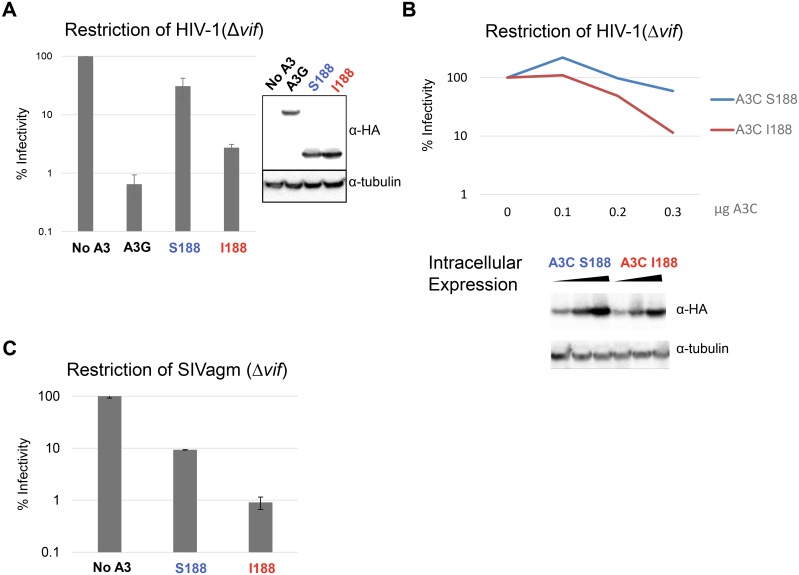
APOBEC3C SNP Isoleucine 188 confers increased antiviral activity. (A) Infectivity of HIV-1 Δ*vif* in the absence of APOBEC3 (No A3), APOBEC3G (A3G), APOBEC3C S188 (A3C S188) and APOBEC3C I188 (A3C I188). 0.3μg of HA-tagged APOBEC3 was expressed in virus-producing cells, and viruses were collected and use for infection. Infectivity in the absence of APOBEC3 is set to 100%. Error bars indicate the standard deviation of triplicate transfections and infections, and this experiment was repeated four times with similar results. Intracellular expression of APOBEC3 was measured by Western Blot using an anti-HA antibody. A section of the blot was probed with an anti-tubulin antibody as a loading control. (B) Dose-response analysis showing restriction of HIVΔ*vif* in the presence of two-fold dilutions of transfected APOBEC3C S188, or APOBEC3C plasmids I188 along with Western blot analysis of APOBEC3C S188, and APOBEC3C I188 protein expression during virus production. This experiment was performed three times, and a representative result is shown. (C) Infectivity of Simian Immunodeficiency virus SIVagmΔ*vif*, in the presence of APOBEC3C S188, APOBEC3C I188. Infectivity is set to 100% for infection with No APOBEC3 present. Error bars indicate the standard deviation of three independent experiments.

Since conserved sequences are often important for function and comparative studies indicate that human APOBEC3C (S188) has weak antiviral/anti-retroelement activity compared to the other human APOBEC3s, we posited that the serine change may contribute to the weak restriction activity of the common variant of APOBEC3C. Therefore, we directly compared APOBEC3C S188 and APOBEC3C I188 for their ability to restrict HIV-1. We transfected the two APOBEC3C variants, S188 and I188, along with VSV-G and an *env*- *vif*- deleted luciferase-expressing HIV-1 provirus (Δ*env*, Δ*vif*). Equal amounts of virus, normalized by p24gag, were subsequently used to infect SupT1 cells and infectivity of the viruses was compared by measuring virus-encoded luciferase. Viral infectivity in the presence of no APOBEC3 is set to 100%. APOBEC3G was used as a positive control because it potently inhibits HIV-1 (Δ*vif*). We found that APOBEC3C I188 restricts infectivity of HIV-1(Δ*vif*) to a level approximately ten-fold greater than the common APOBEC3C, S188, (approx. 30% infectivity versus 3%, respectively) (Fig 3A) even though both proteins are expressed at similar levels. Furthermore, infectivity assays were conducted as a dose-response in the presence of decreasing concentrations of APOBEC3, and the 188 isoleucine variant restricts HIV-1(Δ*vif*) more potently for all conditions (Fig 3B) at similar protein expression levels.

To determine if the APOBEC3C I188 variant has increased potency against another lentivirus, we evaluated its activity against SIVagm (simian immunodeficiency virus that infects African green monkeys). As shown by others, the S188 variant of APOBEC3C restricted infectivity of SIVagm to a greater extent than HIV-1 [9]. However, we found that APOBEC3C I188 restricted SIVagm infectivity ten-fold more than the restriction caused by APOBEC3C S188 (10% versus 1% infectivity, respectively, p<0.05) (Fig 3C). Some APOBEC3s also restrict endogenous retroelements, such as LINE-1s [14, 15]. However, the APOBEC3C I188 variant does not confer increased restriction of LINE-1 as we have previously published [29] and have repeated for this study (S1 Fig). Therefore, the human polymorphism in APOBEC3C at position 188 enhances restriction of at least two primate lentiviruses. Thus, we conclude that a SNP in human APOBEC3C has increased anti-lentiviral activity relative to the APOBEC3C encoded by most humans.

### Isoleucine at position 188 of APOBEC3C enhances enzymatic activity *in vitro*

**Fig 4:**
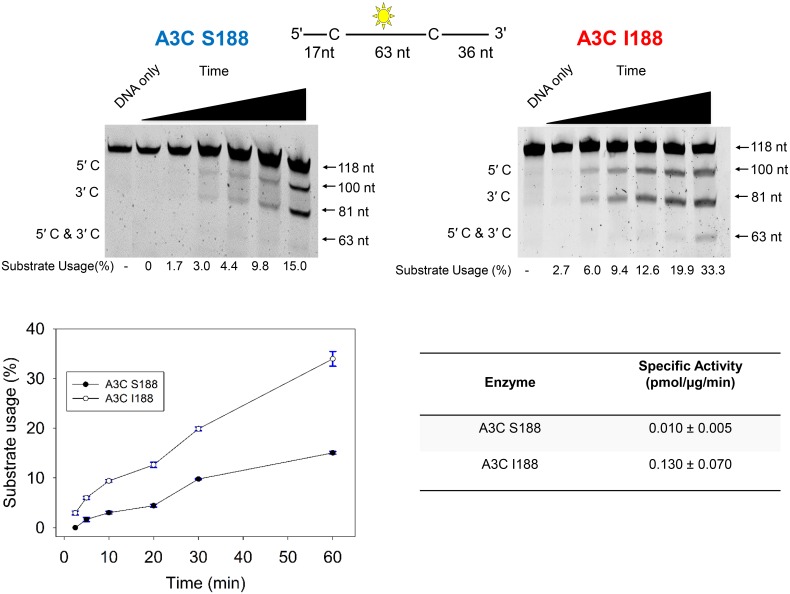
In vitro characterization of APOBEC3C S188 and I188. (Top) The specific activity of APOBEC3C S188 and I188 was determined by incubating the enzyme with a 118 nt ssDNA substrate with an internal fluorescein label (yellow star) and 2 possible sites for cytidine deamination (marked as “C”). Single deaminations of the 5'C and 3'C are detected as the appearance of fluorescently labeled 100 nt and 81 nt fragments, respectively; double deamination of both C residues on the same molecule results in a 63 nt labeled fragment. Substrate usage is quantified for below each lane of the gels. (Bottom) The substrate usage during a 60 min time course was plotted from three independent experiments (bottom left) and used to calculate the specific activity of the enzymes (bottom right).

We wished to investigate whether or not the more potent antiviral activity of APOBEC3C I188 compared to APOBEC3C S188 could be explained by differences in their inherent enzymatic activity. Thus, each protein was produced by expression in a recombinant baculovirus system, purified as described in the Materials and Methods, and tested for its ability to cause cytidine deamination. We examined APOBEC3C S188 and I188 activity using a ssDNA substrate containing two deamination target motifs (Fig 4, top sketch). 5' TTC deamination motifs were used because APOBEC3C preferentially targets this motif [21]. Reactions were carried out as a time-course over 60 minutes and next the substrates were incubated with uracil DNA glycosylase, which modifies uracil-containing DNA and makes it sensitive to cleavage at high pH. Cytidine to uracil mutations leading to DNA cleavage were detected based on a fluorescein label placed between the two deamination motifs (Fig 4, top). Substrate usage was calculated from integrated gel band intensity of cleaved product at either deamination motif relative to the uncleaved substrate (Fig 4). We found that at all time points substrate usage of APOBEC3 I188 was higher than S188, and by 60 minutes I188 had led to twice as many cleavage events as S188 (Fig 4, middle and bottom left). The specific activity of APOBEC3C was determined by calculating the picomoles of substrate used (or deamination events) per microgram of enzyme per minute on a 118 nt ssDNA. The specific activity values were calculated using initial reaction times where the substrate usage was in the linear range (Fig 4, bottom left). We found that APOBEC3C S188 had a specific activity approximately 10-fold lower than I188 (0.010 pmol/μg/min vs 0.130 pmol/μg/min) (Fig 4, bottom right). Therefore, the I188 APOBEC3C more rapidly deaminated cytosines *in vitro* than S188.

Since APOBEC3C I188 has greater cytidine deaminase activity *in vitro* than APOBEC3C S188 (Fig 4), we predicted that it would also have a higher mutational frequency than the APOBEC3C S188. To test this prediction, we used a model *in vitro* system that reconstitutes reverse transcription of RNA to DNA, and observed the ability of APOBEC3 enzymes to induce mutagenesis. The template includes the gene *lacZα*, and blue/white screening was performed to identify mutated reverse transcription products. White colonies, representing templates that were mutated, were then sequenced and the number of mutations induced by each APOBEC3 were quantified. We found that addition of APOBEC3C I188 induced two-fold higher clonal mutation frequency compared to APOBEC3C S188 (S2 Fig, 0.33 x 10^−2^ mutations/bp versus 0.15 x 10^−2^ mutations/bp, respectively). For reactions containing APOBEC3C S188, 100% of clones had zero to one G→A mutation. In contrast, the presence of APOBEC3C I188 caused a noticeable shift in the number of G→A mutations with 32% of clones having more than one mutation and up to four to five mutations in some individual clones (S2 Fig). Overall, isoleucine at position 188 increased the APOBEC3C-induced mutagenesis of ssDNA *in vitro*.

### Dimerization correlates with enhanced antiviral activity of human APOBEC3C

**Fig 5:**
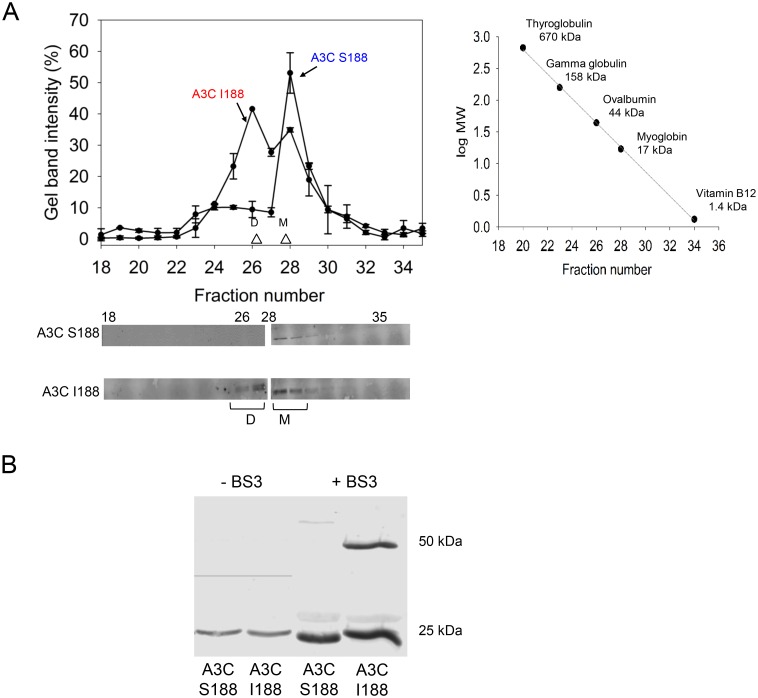
Purified APOBEC3C I188 forms dimers in solution. (A) Size exclusion chromatography profiles of the APOBEC3C S188 and I188 from a 10mL G200 Superdex column were used to calculate the oligomerization states of the enzymes. Molecular weights were calculated by comparing to a calibration curve (see inset on right). When APOBEC3C S188 and APOBEC3C I188 were loaded onto the column, APOBEC3C S188 was a monomer in solution (apparent MW 21 kD in peak fraction), whereas APOBEC3C I188 could form dimers (apparent MW 42 kD in peak fraction) in addition to monomers (apparent MW 21 kD in peak fraction). Chromatograms were made using the integrated gel band intensities from three independent experiments of each protein fraction after resolution by SDS-PAGE. A representative Western blot of the size exclusion chromatography fractions is shown. (B) A3C S188 or I188 were incubated in the absence or presence of 10 μM bis(sulfosuccinimidyl)suberate (BS3) crosslinker (indicated as -BS3 or +BS3) and then visualized through SDS-PAGE and Western blotting. The Western blot demonstrates that A3C S188 remained monomeric in the presence of crosslinker, whereas A3C I188 was both monomeric and dimeric in the presence of the crosslinker. Molecular weight standards are indicated.

Previous studies have reported that the S188 variant of APOBEC3C is a monomeric protein, both in solution [31] and in cells [32]. Indeed, by size exclusion chromatography we also found that baculovirus/*Sf*9-produced APOBEC3C S188 (the common variant) is monomeric (Fig 5A). However, the baculovirus-produced APOBEC3C I188 was in equilibrium between monomer and dimer forms (Fig 5A, apparent molecular weight 21 kDa and 42 kDa, respectively). We confirmed this result using an alternative method of cross-linking the proteins in solution followed by SDS-PAGE and Western blotting. Baculovirus/*Sf*9-produced APOBEC3C S188 or I188 were incubated in the absence or presence of 10 μM bis(sulfosuccinimidyl)suberate (BS3), an amine-amine chemical crosslinker, and then visualized through SDS-PAGE and Western blotting (Fig 5B). A3C S188 remained monomeric in the presence of crosslinker, whereas A3C I188 was partially dimeric in the presence of the crosslinker. The observation that the isoleucine residue at position 188 was able to shift the oligomeric profile of APOBEC3C suggests that residue 188 is important for dimerization. Dimerization has been previously correlated with improved APOBEC3 catalytic activity because it enables efficient scanning of ssDNA to find cytosine targets for deamination [33]. This provides a potential explanation for the increased *in vitro* enzymatic activity of A3C I188.

**Fig 6:**
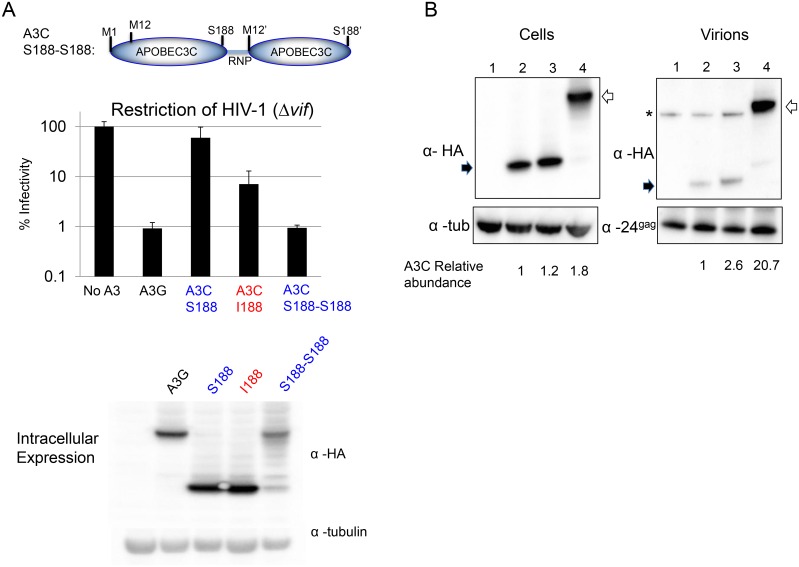
A synthetic dimer of APOBEC3C has increased antiviral activity. (A) Cartoon schematic showing the sequence of the double-domain APOBEC3C. Restriction of HIV-1 Δ*vif* by APOBEC3G(A3G), APOBEC3C S188 (A3C S188), APOBEC3C I188 (A3C I188) and the double-domain APOBEC3C (S188-S188). 0.3μg APOBEC3 was used in this assay. Error bars represent standard deviation of four independent transfections and infections. A Western blot for expression of the APOBEC3 proteins is shown, and is representative of three experiments. (B) Packaging of APOBEC3C variants. Left side: Intracellular expression. Right side: Proteins in the pelleted virions. The intracellular blot was probed with antibody to the HA tag and with antibody to tubulin. The virion pellet blot was probed with antibody to the HA tag and with an antibody to p24gag. A background band in the virion blot with the HA antibody is marked with an asterisk. The single domain APOBEC3C proteins are marked with a solid arrow, while the synthetic double domain APOBEC3C is marked with an open arrow. Relative quantitation of the amounts of APOBEC3C is shown under the panels. An HIV provirus is transfected in each condition; Lanes 1, no APOBEC3; lanes 2, APOBEC3C I188; lanes 3, APOBEC3C S188; lanes 4; the double-domain APOBEC3C (S188-S188).

In order to further test the effects of dimerization of A3C on antiviral activity, we constructed an artificial dimer that consists of two tandem S188 APOBEC3Cs (Fig 6A) and tested the anti-lentiviral activity of this protein. We used the linker that naturally exists between the N- and C-terminal domains of the two double-domain APOBEC3s, APOBEC3D and APOBEC3F, which are the APOBEC3 proteins with the highest sequence identity shared with APOBEC3C. This linker consists of amino acids Arg-Asn-Pro followed by the second APOBEC3 domain starting at Met12 (labeled Met12’ here—see schematic at top of Fig 6A). Western blot analysis shows that this artificial double domain APOBEC3C is expressed in cells and runs at about the same molecular weight as the natural double domain APOBEC3 protein, APOBEC3G (Fig 6A).

We examined the antiviral activity of the synthetic dimer *APOBEC3C* gene (with S188 in both domains, called S188-S188) compared to APOBEC3C S188 and APOBEC3C I188 (Fig 6A) against HIV-1Δ(*vif*). Again, APOBEC3G was used as a positive control. While the APOBEC3C I188 restricted 5–10 fold better than APOBEC3C S188 (Fig 6A: 60% infectivity compared to 8% infectivity), strikingly, APOBEC3C S188-S188 dimer restricted infection as efficiently as APOBEC3G (Fig 5B: approximately 1% infectivity for both conditions). Importantly, the APOBEC3C S188-S188 synthetic dimer restricts infection far greater than two-fold more than the APOBEC3C S188 monomer (Fig 6A: 60% infectivity relative to 1% infectivity), suggesting that the increased antiviral activity is not simply the result of having twice as many active sites. Thus, these results indicate that forced dimerization is sufficient to induce anti-HIV activity of APOBEC3C regardless of the isoleucine at position 188.

In a separate series of experiments, we also examined the ability of each of the A3C variants to be packaged into virions. We found that A3C I188 was not packaged to a greater extent than A3C S188 (compare Fig 6B lanes 2 (A3C I188) to Fig 6B lanes 3 (A3C S188). Thus, the greater activity of A3C I188 correlates better with its increased enzymatic activity than with virion packaging. On the other hand, the synthetic dimer of A3C S188-S188 is packaged into virons 10–20 fold better than the single domain versions of A3C (Fig 6B, lanes 4). This increased packaging could additionally explain the enhanced antiviral activity of the synthetic dimer. This suggests that while natural dimers of APOBEC3C have increased enzymatic activity, a synthetic dimer of an APOBEC3 protein can be created with improved antiviral activity due to increased packaging into virions

### APOBEC3C variants are targeted by Vif

**Fig 7:**
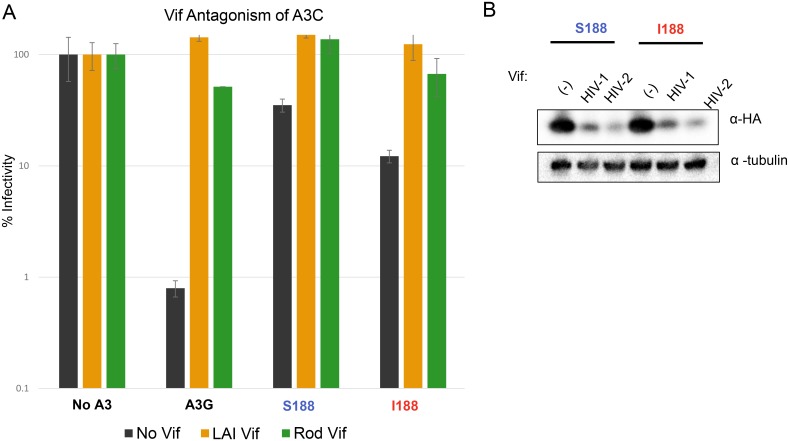
APOBEC3C is targeted by Vif. (A) Restriction of HIV-1 Δ*vif* by APOBEC3C S188 (A3C S188), APOBEC3C I188 (A3C I188), and full recovery of infectivity by the presence of HIV-1 (LAI strain) and HIV-2 (ROD strains) *vif*. 0.4μg of APOBEC3 plasmid was used for each condition, and 0.6μg of each provirus was used. Infectivity of each virus is set to 100% for infection with No APOBEC3 (No A3) present. Error bars represent standard deviation of three independent transfections and infections. Black bars indicate no Vif, orange bars indicate HIV-1 (LAI) Vif, and green bars indicate HIV-2 (ROD) Vif (B) Vif degradation of APOBEC3C S188 and I188 was detected by Western blot analysis. 0.4μg of APOBEC3C and 0.6μg of an HIV-1 provirus (either Δ*vif*, lane labelled as (-)) or containing HIV-1 *vif* or containing HIV-2 *vif*, lanes labelled as HIV-1 or HIV-2) were used to transfect 293T cells and lysates were probed for APOBEC3C expression. Tubulin was used as a loading control.

In the absence of direct clinical or cohort data, we next sought to further evaluate the relevance of APOBEC3C to HIV infection. Previous studies had found that APOBEC3C mRNA is well expressed in primary T cells, which are the target cells of HIV-1 [28]. We further reasoned that if APOBEC3C is indeed a restriction factor relevant to HIV, then one would expect it to be antagonized by the viral Vif protein. To test this, we produced HIV-1 (either lacking *vif*, or expressing either HIV-1 or HIV-2 *vif*) in the presence of APOBEC3C. When we express APOBEC3C I188 during HIV production, the infectivity of the virus is reduced by about ten-fold. However, in the presence of APOBEC3 S188 or I188, both HIV-1 Vif and HIV-2 Vif, restored viral infectivity (Fig 7A). We also conducted western blot analysis to probe for APOBEC3C (S188 and I188) expression in the presence of HIV-1 and HIV-2 Vif proteins (Fig 7B). Consistent with other reports, A3C S188 protein levels are significantly decreased in the presence of HIV-1 Vif [27, 31] as well as HIV-2 Vif [34]. Likewise, the expression of the A3C I188 variant also dramatically decreased in the presence of HIV-1 and HIV-2 Vif. Thus, APOBEC3C I188 effectively antagonized by HIV-1 and HIV-2 Vif which suggests that even the more active form of APOBEC3C in its partial dimer form can still be targeted by both human lentiviral pathogens. These results suggest that APOBEC3C is relevant to HIV infections since Vif has evolved to induce its degradation and APOBEC3C is expressed in HIV target cells.

### APOBEC3C I188 is an ancient human polymorphism that is not found in other hominids

**Fig 8:**
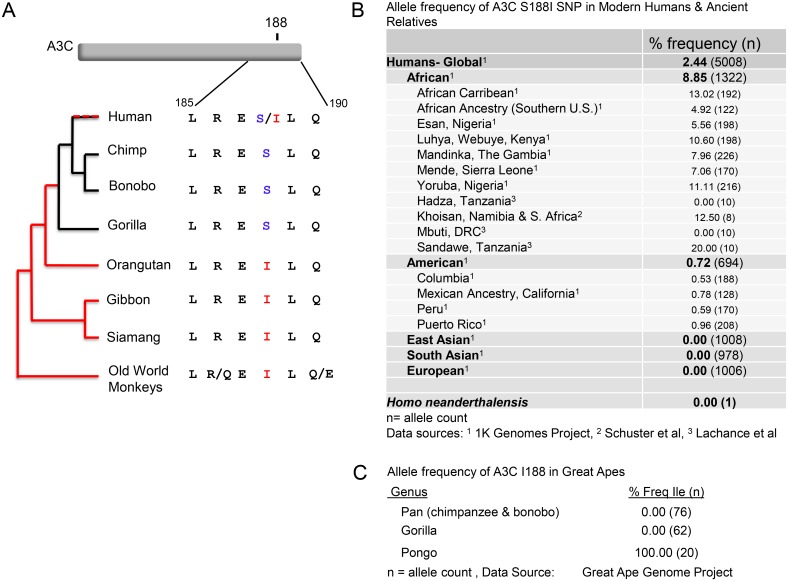
Isoleucine 188 changes to serine in some hominoids but was maintained or reverted back to isoleucine for some human populations. (A) A phylogram of catarrhines, along with an alignment of the C-terminus of APOBEC3C. All old world monkey sequences contained isoleucine at 188 (15 sequences total). (B) Allele frequency of Ile SNP in global human populations, as well as *Homo neanderthalensis*. (C) Allele frequency of Ile 188 in chimpanzees, bonobos, gorillas, and orangutans (n = 79). Sequences were derived from the Great Ape Genome Project[39].

The APOBEC3C I188 variant is present at frequency of 2.4% in the 1000 Genomes Project [29]. Therefore, a relatively small proportion of humans carry a variant of APOBEC3C that is more enzymatically active against lentiviruses. To determine which allele is ancestral at position 188, we constructed a phylogeny of primate *APOBEC3C* sequences. All old world monkeys (N = 15) analyzed encode an isoleucine at position 188 (Fig 8A). Moreover, orangutans, siamangs, and gibbons also encode isoleucine, but the serine change at amino acid 188 occurred in the lineage leading to gorillas, chimpanzees, and humans (Fig 8A). Thus, isoleucine at position 188 is likely the ancestral state, and changed during the evolution of hominids. There are two possible explanations for the existence of the I188 in humans: 1) a reversion back to isoleucine may have occurred in a subpopulation or 2) a polymorphism has been maintained at this site for millions of years, since humans split from their ancestor with gorillas and chimpanzees.

If a serine to isoleucine reversion mutation occurred in recent human evolution, we would expect it to be present only in a limited subset of humans. The frequency of the allele in the 1000 Genomes Project data is 8.9% in populations of African descent, less than 1% frequency in the Americas, and not present in Asia and Europe [29] (Fig 8B). Humans are dramatically more genetically diverse in Africa than on any other continent, therefore we sought to determine if the APOBEC3C I188 allele is distributed across divergent populations in Africa, or if it is present in only a particular subpopulation. The APOBEC3C I188 allele is present in all six African subpopulations analyzed by the 1000 Genomes project, with a frequency ranging between 5.6% and 13% (Fig 8B). However, many of the sub-populations included in the 1000 Genomes Project live in regions affected by the Bantu Expansion, a migration event when Bantu-speaking tribes swept across the continent approximately 3,000 years ago[35, 36]. To determine if the isoleucine allele is present in more diverse African genomes, we determined the *APOBEC3C* sequence from individuals from four hunter-gatherer groups (Hadza, Sandawe, Mbuti, and Khoe-San)[37, 38]. We found that one of the four Khoe-San individuals was heterozygous for the I188 allele, and two out of five Sandawe individuals were heterozygous for the I188 allele (Fig 8B). In conclusion, I188 seems to be a widely distributed SNP in African populations suggesting that the more active allele is very ancient, and may have even been circulating in humans since the birth of the species. Presence of the I188 in the ancient human relative *Homo neanderthalensis* would have provided evidence that the allele has been present in the Homo lineage for at least 600,000 years but we failed to find the I188 SNP in the published Neanderthal genomes.

To determine if other hominoids also possess variation at position 188 we probed the *APOBEC3C* sequences from the Great Ape Genome project[39], and found that none of the great apes included in the study (n = 79) were polymorphic at position 188 (Fig 8C). Ten orangutans were included in the study, and all encoded isoleucine at position 188. In contrast, all gorillas (n = 31), and chimpanzees and bonobos (n = 38), encoded serine at position 188. Humans, gorillas, and chimpanzees diverged from their most recent common ancestor approximately 10 to 20 million years ago[40, 41], and in this ancestral lineage the more active isoleucine allele was lost. However, since some humans express the I188 allele, it is possible S188 never rose to fixation and I188 was maintained as a minor allele for a long period of the evolutionary history of hominoids. Alternatively, it is possible that serine became fixed in the ancestor to gorillas, chimpanzees and humans, but more recently the serine reverted to isoleucine in a subpopulation of humans. Nonetheless, we find that the APOBEC3C I188 is relatively ancient to humans, but is not present to an appreciable extent in out-of-Africa human populations, nor have we found it in other hominids.

## Discussion

APOBEC3C stood out among the seven human APOBEC3 paralogs as it little antiviral or anti-retroelement activity. We observed that the six APOBEC3s with known functions possess a conserved isoleucine at the residue homologous to APOBEC3C position 188, whereas APOBEC3C encodes a serine at this position. However, human APOBEC3C is, in fact, polymorphic at this site, and some humans encode an isoleucine, the residue that correlates with APOBEC3 antiviral/anti-retroelement function. This led us to hypothesize that APOBEC3C may have an as yet overlooked role as a restriction factor, and that the I188 variant may have enhanced antiviral activity compared to the more common variant, S188. *APOBEC3C* has evolved under positive selection in primates and within the interface of binding by the viral protein Vif, suggesting that this gene may have played a role in restriction of lentiviruses over primate evolution. Furthermore, we found that *APOBEC3C* I188 encodes a protein with increased antiviral activity, increased enzymatic activity, and the ability to dimerize in solution. Consistent with this conclusion, an artificial forced dimer of APOBEC3C S188 has vastly increased antiviral activity. We find that the isoleucine at position 188 was lost during hominid evolution but was either reacquired by some humans since humans split with our most recent common ancestor with chimpanzees, or alternatively, has never been lost as an allele and has been maintained as a polymorphism through several million years of hominoid evolution.

### Positive selection of APOBEC3C in primates suggests an ancient role in antiviral defense

Previous studies have shown that APOBEC3C binds to HIV-1 Vif and that E106 is important for Vif binding since mutation to lysine at position 106 completely abrogated HIV-1 Vif binding to APOBEC3C [27, 31]. We found that this residue within the Vif binding interface is evolving under positive selection, and another residue in the Vif-binding region, 77, is also under positive selection. Residue 77 is within the α-2 helix of APOBEC3C, which has also been shown to be important for HIV-1 Vif binding [31]. Additionally, it is possible that Vifs from other lentiviruses target APOBEC3C at different motifs, driving the positive selection in other regions of the protein. For example, APOBEC3C is under positive selection at residues 128 and 130. While these residues are not in the known APOBEC3C:HIV-1 Vif binding interface, the homologous residues of APOBEC3G are involved in HIV-1 Vif binding[42, 43]. Therefore, it is possible that other Vif proteins from other lentiviruses target APOBEC3C at positions 128 and 130, or that ancient lentiviruses have targeted these residues in the past. In summary, rapid evolution of APOBEC3C at the known APOBEC3C:Vif binding interface suggests that APOBEC3C has evolved to block lentiviruses in primates.

### Mechanism of increased activity of APOBEC3C I188 relative to APOBEC3C S188

Our results indicate that the difference in the anti-HIV activity of the APOBEC3C variants S188 and I188 lies in the enzymatic efficiency of the two APOBEC3C proteins. We found that I188 more rapidly deaminates ssDNA *in vitro*. Furthermore, in an *in vitro* RT model system, the presence of APOBEC3C cause a higher mutation frequency than APOBEC3C S188. A previous study correlated multimerization of APOBEC3s with the capacity to restrict lentiviruses [32], and our finding that the monomeric variant (S188) was less antivirally active than the dimer-forming, more active variant (I188), is consistent with this conclusion. Therefore, our model is that isoleucine at position 188 of APOBEC3C enhances lentiviral restriction by improving dimerization and in turn, the enzymatic activity of the protein. One possible reason dimerization is important for APOBEC3C activity, is that it could improve the protein’s ability to scan DNA substrates for cytidine deamination motifs. In fact, I188 lies within α-helix 6, which has been implicated as important for DNA scanning of another APOBEC3, APOBEC3G [44]. The residue at position 188 may not be directly involved in the dimer interface since it is not surface exposed on the crystal structure of A3C I188 (PDB #3VOW)[31]. Nonetheless, our data suggest that the APOBEC3C I188 protein has greater antiviral activity than the more common APOBEC3C protein due to better enzymatic activity that correlates with increased dimerization.

This model that dimerization is a key determinant of APOBEC3C activity is further supported by the fact that a synthetic dimer formed by linking two tandem S188 APOBEC3Cs drastically enhances antiviral activity. In fact, activity is improved even in comparison to I188, the more active variant. I188 only partially dimerizes, and compared to S188 and S188-S188, has an intermediate ability to restrict HIV. Interestingly, the mechanism of increased antiviral activity of A3C S188-S188 is likely due to its increased ability to be packaged into virions. These results suggest that artificial forms of human APOBEC3C proteins can be created that have enhanced antiviral properties that could have therapeutic uses in controlling viral infection.

### Population genetics of human APOBEC3C suggests that the I188 polymorphism is ancient

The isoleucine at position 188 of APOBEC3C is present at approximately 10% frequency across diverse African populations, but almost absent from all other global populations. All human populations outside of Africa are thought to have descended from one or a few migration events out of Africa[45]. As such, humans from non-African populations may lack the APOBEC3C I188 allele because it was excluded in a population bottleneck during the migrations. Or, the allele may have been lost in non-African populations due to drift or a lack of selective pressure. Alternatively, it is possible that loss of the allele was selected for non-African populations. Expression of another APOBEC3, APOBEC3B, has been associated with increased risk of cancer [46, 47]. Therefore, the antiviral function of APOBEC3s may come at an evolutionary trade-off. In fact, this may have driven the maintenance of the less enzymatically active S188 allele for millions of years in humans and ancient human ancestors.

Our phylogenetic analysis shows that APOBEC3C I188 is ancestral in primates, but changed to serine in the clade of apes including gorillas, chimpanzee, and humans. The fact that humans have a polymorphism that corresponds with the ancestral residue could be due to a reversion back to the amino acid present in other primates, but not in gorillas nor chimpanzees. If a reversion occurred it must have happened long ago in human history, since the allele is present in such deeply divergent populations across Africa. However, the allele was likely lost due to a bottleneck in the out-of-Africa populations because it is almost completely missing from non-African populations. Alternatively, it is possible that the isoleucine allele has continued in the human lineage through incomplete lineage sorting (the maintenance of a polymorphism after the divergence of species), since before humans split with their most recent common ancestor with gorillas more than 10 million years ago. Notably, the isloleucine codon, ATT, at position 188 is the same in the human SNP as in all other primates with an Ile at this position in APOBEC3C. While we did not find support for incomplete lineage sorting since we did not find any other hominids that were polymorphic at position 188, the limited number of great ape sequences were included does not allow us to completely rule out this second possibility. Nonetheless, given the increased antiviral activity of APOBEC3C I188 and its fixation in primates other than hominids argues that the gain (or maintenance) of this allele in humans has been driven by a function for protection against pathogens.

### Potential impact on human health

We discovered that an APOBEC3C single nucleotide polymorphism (SNP) that is common in Africa enhances anti-lentiviral activity. This polymorphism may impact human susceptibility to cross-species transmissions of lentiviruses because Vifs from other lentiviruses may not antagonize human APOBEC3C. HIV-1 and HIV-2 Vif are able to antagonize both variants of APOBEC3C so the I188 SNP may not block HIV transmission, so Vif may effectively counteract I188 activity during infection. However, the fact that APOBEC3C is antagonized by Vif does suggest that APOBEC3C is an important barrier that must be countered by the virus during natural infections. Alternatively, it is possible that APOBEC3C antagonism by Vif is an unintended consequence due to Vif binding to another APOBEC3 such as APOBEC3F since APOBEC3C has a Vif binding pocket that is nearly identical to the Vif binding pocket of APOBEC3F[27, 31, 48]. Despite the ability of Vif to antagonize APOBEC3C, it is possible that APOBEC3C I188 still influences HIV susceptibility. In infected individuals possessing the whole APOBEC3 repertoire, Vif has to adapt to counteract multiple antiviral proteins and this may constrain Vif and weaken its activity. In fact, viral genomes sequenced from HIV-1-infected patient cells are extensively mutated by APOBEC3s despite the presence of Vif [49, 50] and the extent of APOBEC3-induced mutagenesis negatively correlates with disease progression rate [51]. As such, it is possible that APOBEC3C I188 may provide some level of protection from HIV transmission or pathogenesis.

## Materials and Methods

### APOBEC3C sequences

APOBEC3C was amplified by RT-PCR from total RNA extracted from chimpanzee, gorilla, orangutan, white-cheeked gibbon, siamang, baboon, sooty mangabey, and red-capped mangabey, and proboscis monkey cells (either fibroblast or lymphoid) obtained from Corriell Repository as well as from the vervet monkey cell line Vero, the tantalus monkey cell line CV-1, and the sabeus cell line V038 provided by the Nonhuman Primate Research Resource (NPRR). Primers were designed to amplify from the 3’ and 5’ UTRs of APOBEC3C mRNA transcripts (5’UTR: CTAAGAGGCTGAACATGAATC’3, 3’UTR: 5’GGCTAGAGGAGACAGACCATGA’3). The APOBEC3C amplicons were cloned into pGEM vectors, and then sequenced. The S188-188 forced dimer was designed to mimic the linker between the two domains of the double-domain APOBEC3F. The N-terminal subunit consists of APOBEC3C residues 1–189 (residue 190 is removed), followed by the residues RNP, which serve as a linker. The C-terminal APOBEC3C begins at the second start codon, M12. The dimer S188-S188 APOBEC3C was constructed by overlap extension PCR. Two separate PCRs were performed for the N terminal and C terminal APOBEC3C subunits (1^st^ domain, For: TTCAGGATCCATGAATCCAGAGATC, 1^st^ domain, Rev: GCCTCCATTGGGTCCCGGAGACTCTCCCGTAGCCTTCTTT, 2nd domain, For: TCCAGGATCCATGAATCCACAGATC, 2^nd^ Rev: GCCCTCTAGATTAGGCGTAGTCAGG), and these amplicons were annealed in a third PCR reaction using the 1^st^ domain For and the 2^nd^ domain Rev primers.

### Sequence analysis

*APOBEC3C* genes were aligned using Geneious software. To test for positive selection, maximum likelihood tests were performed using the PAML statistical software suite [52]. The *APOBEC3C* genes were subjected to tests that allowed for positive selection (M8 model), or disallowed positive selection (M8a model). The analyses were performed with the F3X4 codon model, and multiple starting omega values were used, ranging between 0.5 and 1.4. Specific residues with signatures of positive selection with a posterior probability of 99% or greater were identified by Bayes Empirical Bayes analysis. Ancestral APOBEC3C sequences were reconstructed by the likelihood/Empirical Bayes approach using the codeml program in PAML. Brach analysis to identify particular primate branches with signatures of positive selection in APOBEC3C were performed in two ways. Overall dN/dS values were calculated with PAML, using the free ratio model. Additionally, a branch-site test to identify statistically significant signatures of episodic selection was performed using the Branch-site REL method in the HyPhy software suite [53].

### APOBEC3, provirus, and LINE-1 plasmids

APOBEC3Cs were cloned into the BamHI and XhoI sites of pCDNA3.1 by PCR addition of restriction sites (BamHI and XhoI) to the N and C termini of APOBEC3C. The human APOBEC3C plasmid we previously obtained from the AIDS Repository contained the SNP rs11551111, which is not common (no reported frequency according to dbSNP). Therefore, we used site-directed mutagenesis to change the asparagine at position 31 to aspartic acid (For: GCCAACGATCGGGACGAAACTTGGC, Rev: GCCAAGTTTCGTCCCGATCGTTGGC). A hemagglutinin tag was inserted into the XhoI and XbaI sites of pCDNA3.1, at the C-terminus of each APOBEC3C sequence. APOBEC3G and APOBEC3A were also in a pCDNA3.1 backbone, with a Kozak sequence, as well as a hemagglutinin tag at the N-terminus. HIVΔenv,Δ*vif*, HIVΔ*vif* + HIV-1 *vif*, HIVΔ*vif* +HIV-2 *vif* have been described elsewhere [54]. SIVagm Δ*env*, Δ*vif* was kindly provided by Nathaniel Landau.

### Infectivity assays

Single round HIV-1 and SIVagm infectivity assays were performed as previously described [55]. 293T cells (American Type Culture Collection) were plated at a density of 5 X 10^3^ cells per well of a 24-well plate. The next day, the cells were transfected with 0.3μg provirus encoding luciferase as a marker gene 0.1μg pL-VSV-G, and 0.3μg pCDNA3.1.APOBEC3.HA or empty pCDNA3.1 plasmid. For the dose response infectivity assay, either 0.1 μg, 0.2μg, or 0.3μg APOBEC3 plasmid was used. For experiments involving Vif expression, 0.2μg of APOBEC3 was used. Forty-eight hours after transfection, virions were harvested. For SIVagm infectivity assays, SupT1 cells were infected with 10μl of each virus and treated with 20μg/ml DEAE/dextran. For HIV infectivity assays, ELISA was performed to quantify p24, and virus equivalent to 2ng p24 was used for infections. For all infectivity assays, 5 X 10^4^ were infected in a 96 well dish. Seventy-two hours later, infected cells were lysed in luciferase lysis reagent (Brightglo, Promega) and luciferase expression was measured on a luminometer (LUMISTAR Omega, BMG). Infectivity of each virus was compared by setting infectivity of the “No APOBEC3” control to 100%. All HIV-1 constructs are based on the LAI strain.

### LINE-1 assays

To assay for restriction of LINE-1 retrotransposition 293T cells were transfected with 200ng LINE-1 plasmids pYX016 and pYX015[56], along with 100ng of APOBEC3C S188 or I188, APOBEC3C, 10ng APOBEC3A, or empty pCDNA3.1 plasmid. The next day, the cells were treated with 2.5 ug/ul puromycin to select for transformants. Three days later, expression of renilla and firefly luciferase were assayed using a luminometer. The LINE-1 plasmids encode firefly luciferase disrupted by a splice site, so expression only occurs after retrotransposition, whereas renilla luciferase expression is not dependent upon retrotransposition. Percent retrotransposition is reported by setting retrotransposition (firefly luciferase values divided by renilla luciferase values) in the absence of APOBEC3 to 100%.

### Intracellular protein expression and packaging

Intracellular expression of the APOBEC3 proteins during virion production was evaluated by lysis of the virion-producing 293T cells with Radio Immunoprecipitation Assay buffer (RIPA), with protease inhibitor (50mM Tris, 150mM sodium chloride, 0.1% SDS, 0.5% sodium deoxycholate, 1% NP-40, protease inhibitor cocktail cOmplete by Roche). Lysates were resolved on an SDS-PAGE gel in MES buffer, and transferred to a PVDF membrane for Western blot analysis, using and anti-HA (BioLegend) antibody and anti-tubulin (Sigma-Aldrich) antibody. Endogenous levels of APOBEC3C were measured by Western blotting with antibody purchased from Fisher (product # PA5- 27629). HRP-conjugated secondary antibodies (Santa Cruz) were used to detect primary antibodies.

Packaging of APOBEC3 into virions was evaluated by co-transfection of 100 ng of each APOBEC3 expression plasmid with 500 ng of an HIV proviral clone (LAI) containing a deletion in *vif* in each well of a 12-well plate. Three days after transfection, 1 ml of supernatant was collected, filtered through a 0.2 micron filter, and concentrated by pelleting in a microcentrifuge at 13K rpm for 60 minutes and resuspended in 80 μl. The amount of p24gag was determined by ELISA (Advanced Bioscience Laboratories). Equal quantities of p24gag were lysed and run on an SDS-PAGE gel. The Western blots were probed with an anti-HA antibody for A3C protein and with a p24gag antibody for virus production and HRP-conjugated secondary antibodies were used to detect primary antibodies. Cells were lysed as described above. The chemiluminescent signals from the Western blots were imaged using a ChemiDoc MP Imaging System (Bio-Rad) and quantified in the linear detection range.

### Recombinant protein expression and purification

Recombinant baculovirus production for APOBEC3C S188 was carried out in the pACG2T transfer vector (BD Biosciences), as described previously [57]. Recombinant baculovirus production for APOBEC3C I188 was carried out in the pFastbac1-GST-APOBEC3C vector according to the Bac-to-Bac expression system (Life Technologies) and as described previously [58]. Recombinant virus was then used to infect *Sf9* cells. Cells were harvested 72 hours after infection, lysed, treated with RNaseA, and clarified cell lysates were incubated with glutathione-sepharose 4B resin (GE Healthcare) at 4°C and subjected to a series of salt washes, as described previously[59]. The APOBEC3C S188, APOBEC3C I188 enzymes were eluted from the glutathione-sepharose resin (GE Healthcare) with the GST tag, as previously described [59]. The samples were then treated with thrombin (GE Healthcare) for 6 hr at 21°C to cleave the GST tag.

### Size exclusion chromatography

The oligomerization states of the APOBEC3C enzymes were determined by loading 10 μg of purified enzyme on a 10 mL Superdex 200 (GE Healthcare) size exclusion column. The column was prepared by pouring the resin bed in a column with 16-cm height and 0.5-cm diameter. The running buffer contained 50 mM Tris pH 8.0, 200 mM NaCl and 1 mM DTT. The Bio-Rad standard set was used to generate a standard curve from which molecular masses and oligomerization states of the enzymes were determined.

### Protein crosslinking by BS3

A3C S188 and A3C I188 (0.5 μM) were incubated with 10 μM BS3 in 20 mM Hepes (pH 7.5), 150 mM NaCl and 1 mM DTT for 1 hour at 21°C. Crosslinked proteins were resolved on a 12% SDS-PAGE gel, transferred to a nitrocellulose membrane for Western Blot analysis and visualized using primary antibody for native APOBEC3C (GeneTex) and secondary IRdye labeled goat anti-rabbit antibody compatible with the LI-COR/Odyssey system.

### *In vitro* deamination assay

All ssDNA substrates were obtained from Tri-Link Biotechnologies as previously published [44]. Reactions were carried out under single-hit conditions (*i*.*e*. <15% substrate usage) to ensure that a single enzyme carried out the deaminations on the ssDNA[60]. A ssDNA substrate containing two 5′-TTC motifs (100 nM) was incubated with 350 nM of APOBEC3C I188 or 700 nM of APOBEC3C S188 for 5 to 30 min at 37°C in RT buffer (50 mM Tris, pH 7.5, 40 mM KCl, 10 mM MgCl_2_, and 1 mM DTT). The reaction time was varied on each ssDNA according to the specific activity of the enzymes to ensure <15% substrate usage. Reactions were started by the addition of the ssDNA substrate. APOBEC3C-catalyzed deaminations were detected by treating the ssDNA with uracil DNA glycosylase (New England Biolabs) and heating under alkaline conditions before resolving the fluorescein-labeled ssDNA on 10 or 20% (v/v) denaturing polyacrylamide gels, depending on the sizes of the ssDNA fragments. Gel photos were obtained using a Typhoon Trio multipurpose scanner (GE Healthcare), and integrated gel band intensities were analyzed using ImageQuant (GE Healthcare). The specific activity was calculated from single-hit condition reactions by determining the picomoles of substrate used per minute for a microgram of enzyme.

### In vitro reverse transcription assay

Mutagenesis of ssDNA by A3 enzymes during reverse transcription of an RNA template was assessed using an *in vitro* assay, which models reverse transcription of an RNA template and second-strand synthesis. The method is described in detail in Feng and Chelico 2011 [33]. This system uses an *in vitro* synthesized RNA, which contains a polypurine tract (PPT), a protease gene (prot) of HIV, and a lacZα reporter for blue/white screening. The RNA is reverse transcribed to (−)DNA by reverse transcriptase (RT) by annealing a DNA primer and after the RNaseH domain of RT removes the RNA, the PPT enables second-strand (+)DNA synthesis by acting as a primer. A 368-nt RNA template (50 nM) is annealed to a DNA primer (24-nt) and incubated with 1.5 μM of nucleocapsid (NC), 1.2 μM of reverse transcriptase (RT) and 500 μM of dNTPs in RT buffer in the presence or absence of 350 nM of each APOBEC3C enzyme. The RNA template contained an HIV-1 PPT, nucleotides (nt 2282–2401) from the HIV-1 clone 93th253.3 (accession number U51189), and *lacZα*. The resulting dsDNA that is synthesized from this *in vitro* system was PCR amplified using Pfu C_x_ Turbo Hotstart (Agilent Technologies) that can use uracils as a template with high fidelity. These amplicons were then cloned into a pET-Blue vector backbone that allows for blue-white screening of the synthesized *lacZα*. At least twenty-five mutated clones for each condition were tested.

### SNP analysis

1000 Genomes Project data was mined for the presence of SNPs at position 188 of APOBEC3C (SNP ID rs112120857). To further elucidate the frequency of the APOBEC3C I188 SNP across Africa, we analyzed the genomes reported by Schuster et al.[38] and Lachance et al.[37] for the presence of the I188 allele. To assay for the presence of SNP at position 188 in other hominoids, we mined the Great Ape Genome Project [42] (accession number SRP018689) sequences in the NCBI short read archive.

## Supporting Information

S1 Fig Inhibition of Line-1 replication by APOBEC3A and S188 and I188 APOBEC3C. Ten times more APOBEC3C was used in this assay than APOBEC3A. The Line-1 plasmid constitutively expresses renilla luciferase, and only expresses firefly luciferase upon retrotransposition. Values are shown as the ration of firefly luciferase expressed over renilla luciferase expression. Averages of three replicates are shown and this experiment was repeated twice.
(TIFF)

S2 Fig *In vitro* reverse transcription assay. An in vitro HIV replication assay was utilized to determine the APOBEC3C enzyme ability to catalyze deaminations during proviral DNA synthesis. This system reconstitutes reverse transcription of (−)DNA and synthesis of (+)DNA by using a substrate which contains a polypurine tract (PPT), 120-nt of the protease gene (prot) of HIV, and a lacZα reporter. (Left) G→A mutations are scored for each clone and mutational spectra are plotted as the percentage of clones containing a mutation at a particular location (nt) in the 368 nt prot-lacZα construct. The number of mutations per base pair for each APOBEC3C is indicated above the spectra. (Right) Histograms depicting the population distribution of mutations per *prot-lacZ*α for the APOBEC3C enzymes.
(TIFF)
