# Supplementary figures and images for: A Single Nucleotide Polymorphism in Human APOBEC3C Enhances Restriction of Lentiviruses
Source: PLoS Pathog. 2016 Oct 12;12(10):e1005865. doi: 10.1371/journal.ppat.1005865 (PMC5061367; doi:10.1371/journal.ppat.1005865)

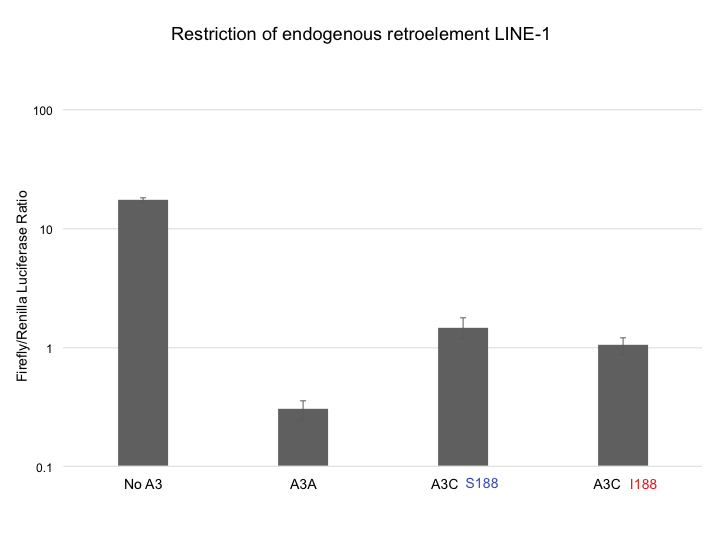

Supplement: S1 Fig — Ten times more APOBEC3C was used in this assay than APOBEC3A. The Line-1 plasmid constitutively expresses renilla luciferase, and only expresses firefly luciferase upon retrotransposition. Values are shown as the ration of firefly luciferase expressed over renilla luciferase expression. Averages of three replicates are shown and this experiment was repeated twice. (TIFF) [file ppat.1005865.s001.tiff]

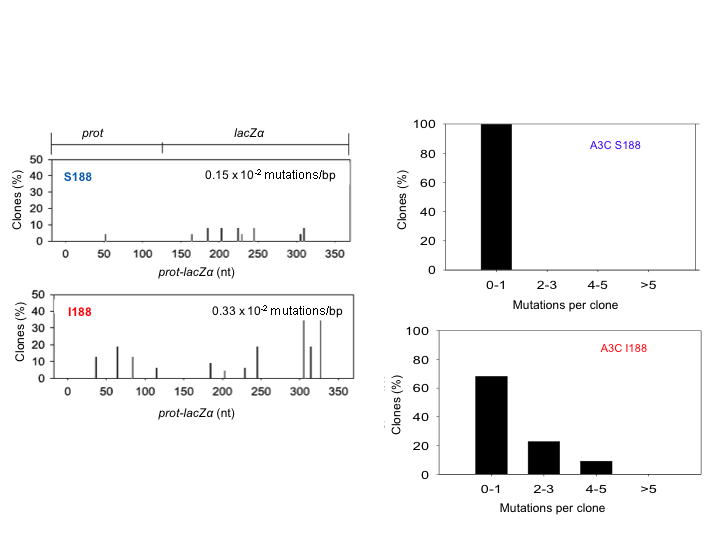

Supplement: S2 Fig — An in vitro HIV replication assay was utilized to determine the APOBEC3C enzyme ability to catalyze deaminations during proviral DNA synthesis. This system reconstitutes reverse transcription of (−)DNA and synthesis of (+)DNA by using a substrate which contains a polypurine tract (PPT), 120-nt of the protease gene (prot) of HIV, and a lacZα reporter. (Left) G→A mutations are scored for each clone and mutational spectra are plotted as the percentage of clones containing a mutation at a particular location (nt) in the 368 nt prot-lacZα construct. The number of mutations per base pair for each APOBEC3C is indicated above the spectra. (Right) Histograms depicting the population distribution of mutations per prot-lacZα for the APOBEC3C enzymes. (TIFF) [file ppat.1005865.s002.tiff]
